# Chemical constituent characterization and determination of *Quisqualis fructus* based on UPLC-Q-TOF-MS and HPLC combined with fingerprint and chemometric analysis

**DOI:** 10.3389/fpls.2024.1418480

**Published:** 2024-06-21

**Authors:** Lin Yang, Lei Dai, Weihan Qin, Yiwu Wang, Jianing Zhao, Shuxiang Pan, Dan He

**Affiliations:** ^1^ Chongqing Pharmaceutical Preparation Engineering Technology Research Center, Chongqing Medical and Pharmaceutical College, Chongqing, China; ^2^ Chongqing Research Center for Pharmaceutical Engineering, College of Pharmacy, Chongqing Medical University, Chongqing, China; ^3^ Medicinal Chemistry Institute of Traditional Chinese Medicine, Chongqing Academy of Chinese Material Medica, Chongqing, China

**Keywords:** *Quisqualis fructus*, UPLC-Q-TOF, HPLC, chemometric, fingerprint

## Abstract

*Quisqualis fructus* (QF) is a traditional Chinese medicine (TCM) that it has a long history in the therapeutic field of killing parasites, eliminating accumulation, and stopping diarrhea. However, the therapeutic material basis of QF is remaining ambiguous nowadays. The geographical origin differences of QF are also usually ignored in the process of medication. In this study, the alcohol–aqueous soluble constituents in QF from different origins were systematically characterized and accurately measured by ultra-high performance liquid chromatography coupled to quadrupole-time-of-flight mass spectrometry (UPLC-Q-TOF-MS) and high-performance liquid chromatography (HPLC) respectively. Chemometric analysis was performed for origin differentiation and screening of potential quality marker (Q-marker). Finally, A total of 106 constituents were tentatively characterized in positive and negative ion modes, including 29 fatty acids, 26 organic acids, 11 amino acids and derivatives, 10 glycosides, 9 alkaloids and derivatives, and 21 other constituents. QF from different origins were effectively distinguished and 16 constituents were selected as the potential Q-markers subsequently. Four representative components (trigonelline, adenosine, ellagic acid, and 3,3’-di-O-methylellagic acid) in QF samples were simultaneously determined. HPLC fingerprint analysis indicated that the similarity between 16 batches of QF was in the range of 0.870–0.999. The above results provide some insights for the research on the pharmacodynamic constituents, quality control, and geographical discrimination of QF.

## Graphical Abstract

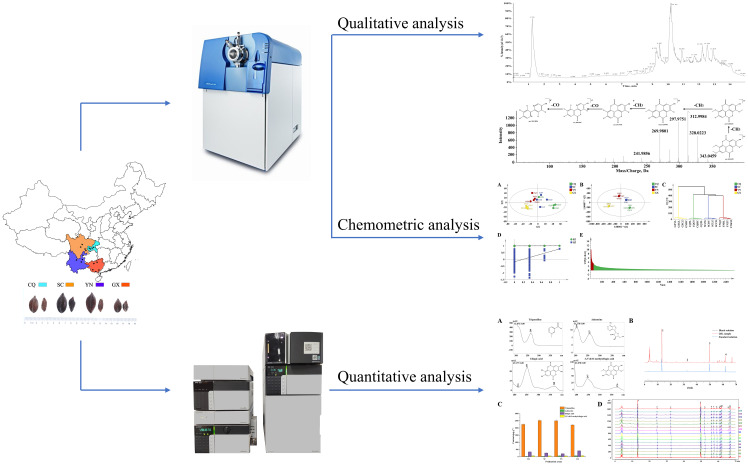

## 1 Introduction

*Quisqualis fructus* (QF) is a dried ripe fruit belonging to the combretum family with an oval shape and five longitudinal edges, 2.5–4 cm long and approximately 2 cm in diameter, dark brown to purple-black on the surface. It has the following functions: killing parasites and eliminating accumulation, strengthening the spleen, and stopping diarrhea in the clinic (China Pharmacopoeia [Part I], 2020). Modern pharmacological research revealed that the alcohol extract of QF has insecticidal properties such as anti-mosquito, anti-silkworm, and anti-Giardia lamblia (Govindarajan et al., 2016; Cao et al., 2023), as well as antibacterial (Agarwal et al., 2017) and antioxidant (Rastogi et al., 2019) characteristics; it can also inhibit liver cancer cell proliferation (Song et al., 2021) and improve benign prostatic hyperplasia (Kim et al., 2020). It is one of the clinical prescription ingredients that include 50 kinds of Chinese patent medicine prescriptions and 166 kinds of herbal prescriptions. However, research on the pharmacodynamic constituents, quality control, and origin differences of QF is relatively rare by retrieving relevant databases such as PubMed, CNKI, and Web of Science (Yaozh *Traditional Chinese medicine of* Quisqualis indica L., 2024).

The medical books in past dynasties recorded that QF originated in India. It was first recorded in “southern grasses and trees” of the Jin Dynasty with the name of “Liu Qiu Zi” (Wang et al., 2015). It was one of the major genuine medicinal materials in Chongqing China according to the fourth national survey of traditional Chinese medicine (TCM) resources, and also widely distributed in the southwest regions such as Sichuan, Yunnan, and Guangxi provinces in China (Zhong et al., 2020). Genuine medicinal materials is usually selected based on the quality standard, whereas research on relevant material basis and quality evaluation of QF is deficient both domestically and globally all the time (Luo et al., 2021). In terms of the qualitative analysis aspect, a previous study reported that nine constituents including 3,3’-di-O-methylellagic acid, 3,3’,4’-tri-O-methylellagic acid, and others were isolated and identified from the ethanol extract of QF by traditional separation and purification (Zhang et al., 2015). It is not neglected that the above approach has limitations such as cumbersome operation and incomplete identification. In terms of the quantitative analysis aspect, trigonelline and quisqualic acid in QF were determined simultaneously by ultra-hydrophilic interaction chromatography-tandem mass spectrometry (UHILIC-MS/MS). They were also determined individually by high-performance liquid chromatography (HPLC) through pre-column derivatization (China Pharmacopoeia [Part I], 2020; Liao et al., 2021; Wang et al., 2023), while the single constituent is monotonous to comprehensively reflect the quality of QF.

Ultra-high-performance liquid chromatography coupled with quadrupole time-of-flight mass spectrometry (UPLC-Q-TOF-MS) combines the efficient and rapid separation ability of chromatography, as well as the accurate and sensitive qualitative and quantitative ability of mass spectrometry. With the advantages of high sensitivity and high resolution, a scanning speed of microseconds, and a wide range of quality detection, it was widely utilized to identify chemical constituents, evaluate quality, and elucidate the pharmacodynamic mechanism of TCM (Ma et al., 2022; Yang et al., 2023). As a significant data processing means for quality control and authentication of various herbs, chemometrics could visualize the repetitive data by explaining and simplifying the large amount of data information generated by high-throughput mass spectrometry (Rebiai et al., 2022). Furthermore, the fast and convenient approach of HPLC has also played a vital role in the quality evaluation of TCM during the past few decades (Ren et al., 2020).

In this study, the chemical constituents including 16 batches of QF samples from four main producing areas were identified and parsed by UPLC-Q-TOF-MS. Subsequently, chemometrics was employed to screen potential Q-markers and compare the geographical differences of QF from different origins. In combination with the above qualitative result, a rapid and convenient reversed-phase HPLC method was established to simultaneously determine the four constituents of QF, namely, trigonelline, adenosine, ellagic acid, and 3,3’-di-O-methylellagic acid. Meanwhile, fingerprints were utilized to evaluate the similarity of QF between 16 batches. Overall, the above research comprehensively elucidated the therapeutic material basis of QF by qualitative analysis and effectively distinguished the origin differences by chemometric analysis. The established quantitative analysis approach could be widely used for quality evaluation in the future.

## 2 Materials and methods

### 2.1 Chemicals and materials

Acetonitrile (LC-MS grade) and methanol (HPLC grade) were purchased from Merck (Darmstadt, Germany). Formic acid (HPLC grade) and sodium 1-octane sulfonate (HPLC grade) were purchased from Shanghai Mackin Biochemical Co., Ltd (Shanghai, China). Phosphoric acid (HPLC grade) and other analytical reagents (AR) were obtained from Chuandong Chemical Group Co., Ltd (Chongqing, China). Distilled water was obtained by a Milli-Q water system (Millipore, Bedford, MA, USA). Trigonelline (315D021), ellagic acid (P1884386), and 3,3’-di-O-methylellagic acid (P2775319) were purchased from Shanghai Taitan Bio-Technology Co., Ltd (Shanghai, China). Adenosine (110879–200202), arginine (140685–202209), glutamic acid (140690–202305), palmitic acid (190029–201904), and myristic acid (190162–201501) were purchased from the National Institutes for Food and Drug Control. The purity of all compounds was not less than 98%.

### 2.2 Plant materials and sample preparation

A total of 16 samples of QF from Chongqing (S1–S4), Sichuan (S5–S8), Yunnan (S9–S12), and Guangxi (S13–S16) were purchased from native herb markets and drug retail stores in various producing areas. It was identified as the dry and mature fruit of *Quisqualis fructus* by Associate Researcher Qin Weihan (Chongqing Institute of Traditional Chinese Medicine).

### 2.3 Standard solutions and sample preparation

The standard stock solutions of trigonelline, ellagic acid, 3,3’-di-O-methylellagic acid, adenosine, arginine, glutamic acid, palmitic acid, and myristic acid were made by accurate weight and individual dissolution in methanol. To obtain a series of working standard solutions, the above stock solutions were mixed and diluted to the appropriate concentration in methanol gradually. Finally, all solutions were stored at 4°C until analysis.

According to the extraction method of Chinese Pharmacopoeia (China Pharmacopoeia [Part I], 2020), the QF samples were pulverized as powder and passed through an 80-mesh sieve for subsequent utilization. The powder (0.5 g) was accurately weighed and ultrasonically extracted in 80% methanol (5 mL) for 30 min (250 W, 40 kHz). It was centrifuged at 10,000 rpm for 5 min and passed through a 0.22-μm microporous membrane for HPLC analysis. Furthermore, the above solutions were used for UPLC-Q-TOF analysis after 20 times dilution. Moreover, blank solution was prepared in the same way for the deduction of background interference. Mixing aliquots of each sample was taken as quality control (QC) sample, and it was used to investigate the stability and repeatability every six sequence samples.

### 2.4 UPLC-QTOF-MS conditions

A Shimadzu LC 20AD_XR_ UPLC system (Kyoto, Japan) coupled with an ACE Excel 3 Super C18 column (100 mm × 2.1 mm, 3.0 μm) was used for chromatographic separation. The mobile phase was 0.1% formic acid in water (A) and acetonitrile (B) with a flow rate of 0.25 mL/min. The injection volume was 2 μL. The conditions of gradient elution were as follows: 0–1.2 min, 6% B; 1.2–9.5 min, 6%–75% B; 9.5–11 min, 75%–90% B; 11.0–13 min, 90% B; 13–14 min, 90% B; 14–15 min, 6% B.

Mass spectrometry was performed on an AB SCIEX Q-TOF 5600 mass spectrometer (Foster City, CA, USA) with an ESI source. Information-dependent acquisition (IDA) of ions was employed in both positive and negative ion modes with the mass range of 100–1,000 *m*/*z*. The ion temperature was 600°C with the 5,500 V and −4,500 V of spray voltages. The ion source gas 1 (GS1), ion source gas 2 (GS2), and curtain gas (CUR) were 55 psi, 55 psi, and 25 psi, respectively. The declustering potential, collision energy, and collision energy spread were 100 eV, 40 eV, and 15 eV, respectively. Multiple mass defect function and dynamic background subtraction were the conditions to trigger the second stage and gave priority to secondary scanning.

### 2.5 HPLC conditions

A Shimadzu LC 20AT HPLC system coupled with an SPD-M40 detector (Kyoto, Japan) was used for quantitative analysis. The column of Thermo GOLD C18 (250 mm×4.6 mm, 5 μm) was maintained at 30°C. The flow rate of the mobile phase was 1 mL/min with 0.1% phosphoric acid–10 mmol/L sodium 1-octane sulfonate in water (A) and acetonitrile (B). The conditions of gradient elution were as follows: 0–20 min, 3%–8% B; 20–30 min, 8%–10% B; 30–45 min, 10%–15% B; 45–60 min, 15%–40% B; 60–70 min, 40% B. The injection volume and detection wavelength were 20 μL and 254 nm, respectively.

### 2.6 Data processing and analysis

Raw UPLC-QTOF-MS files of each batch were imported into Peakview1.2 software for self-constructed database comparison, which was constructed according to relevant literature, including chemical names, molecular formula, and CAS numbers. After converting the raw UPLC-QTOF-MS files from wiff to abf format by the ABF converter, each raw file was imported into MS Dial for public database comparison (http://prime.psc.riken.jp/compms/msdial/main.html#MSP), including peak extraction, peak recognition, peak alignment, setting of addition ion, and importing database.

Chemometric analysis was performed on SIMCA14.1 software (Umetrics AB, Umea, Sweden). To obtain the three-dimensional matrix data [including sample name, retention time-mass charge ratio (*t*_R_-m/z), and peak intensity] of UPLC-QTOF-MS for chemometric analysis, peak extraction, peak alignment, peak matching, and normalization were performed by using Notepad software. The similarity evaluation was performed on the Similarity Evaluation System for Chromatographic Fingerprint of Traditional Chinese Medicine (2012 Edition).

## 3 Results and discussion

### 3.1 Qualitative analysis of QF from different origins by UPLC-Q-TOF-MS

**Figure 1:**
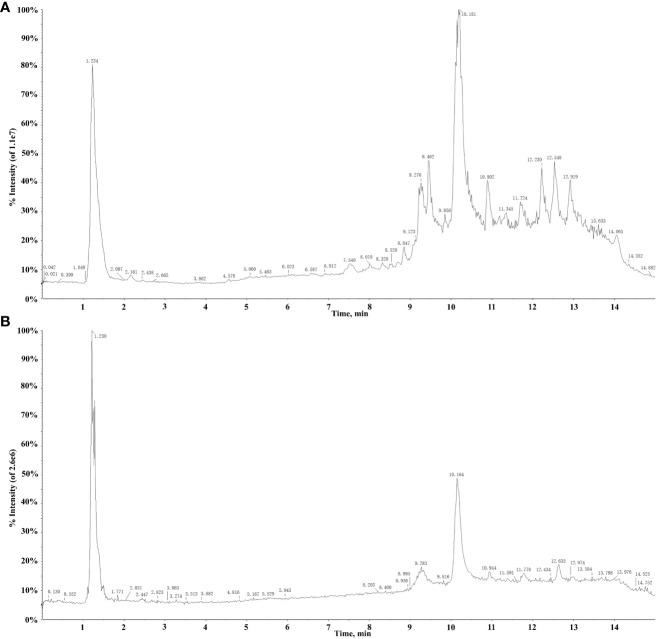
TICs of QF in the positive **(A)** and negative **(B)** mode.

The extract solutions of QF were comprehensively identified through UPLC-Q-TOF-MS. The total ion chromatograms (TICs) in positive and negative ion mode are displayed in **Figure 1**. Meanwhile, the chemical constituents containing secondary fragment ions were analyzed and processed in Peakview1.2 and MS Dial ver.5.1 software. A total of 106 constituents were identified and characterized through the self-constructed database, public database, relevant literature, and reference standard, namely, 29 fatty acids, 26 organic acids, 11 amino acids and derivatives, 10 glycosides, 9 alkaloids and derivatives, and 21 other compounds. Among them, 68 constituents were first characterized through public database matching because of the more sensitive detection methods and comparative analysis of multiple producing areas. Meanwhile, 30 constituents were consistent with previous literature report and were further proved to be present in QF. Furthermore, eight constituents were first characterized through reference standards to provide a scientific basis for the identification of QF. The detailed information is listed in **Table 1**.

**Table 1:** The identified chemical constituents of QF by UPLC-Q-TOF-MS.

No.	Constituent name	Rt (min)	Ion type	Formula	Observed *m/z*	Error (ppm)	MS/MS fragment (*m/z*)
1	Choline^1^	1.11	[M]^+^	C_5_H_14_NO	104.10699	−0.4	104.1054, 60.0803, 58.0648
2	L-Histidine^1,2^	1.16	[M+H]^+^	C_6_H_9_N_3_O_2_	156.07675	0.1	110.0713, 93.0449, 83.0605, 56.0496
3	L(+)-Arginine^1,2,3^	1.16	[M+H]^+^	C_6_H_14_N_4_O_2_	175.11895	0	70.0652, 60.0554
4	L-Carnitine^1^	1.19	[M+H]^+^	C_7_H_15_NO_3_	162.11247	1.3	102.0894, 85.0279, 58.0644
5	Mannitol^1^	1.2	[M-H]^-^	C_6_H_14_O_6_	181.07176	2.6	101.0259, 89.0257, 71.0140, 59.0136
6	Glutamic acid^1,2,3^	1.21	[M+H]^+^	C_5_H_9_NO_4_	148.06043	−0.2	84.0440, 56.0479
7	Stachyose^1,2^	1.22	[M-H]^-^	C_24_H_44_O_22_	683.22515	0.1	341.1068, 179.0558, 161.0450
8	L-Tyrosine^1,2^	1.23	[M+H]^+^	C_9_H_11_NO_3_	182.08117	−1.6	136.0761, 123.0432, 91.0527, 77.0381
9	L-Asparagine^1,2^	1.23	[M+H]^+^	C_4_H_8_N_2_O_3_	133.06077	−1.2	133.0608
10	L-Pyroglutamic acid^1^	1.23	[M+H]^+^	C_5_H_7_NO_3_	130.04987	0.4	84.0433, 56.0491
11	Trigonelline^1,2,3^	1.24	[M+H]^+^	C_7_H_7_NO_2_	138.05496	0.7	92.0489, 78.0337, 65.0385, 51.0228
12	Phenolic glycosides^1^	1.25	[M-H]^-^	C_18_H_18_O_9_	377.08781	−4.8	341.1076, 215.0319, 179.0558, 119.0348, 89.0240
13	Gluconic acid^1^	1.25	[M-H]^-^	C_6_H_12_O_7_	195.05103	3.6	129.0206, 99.0100, 87.0102, 75.0095
14	L-Proline^1,2^	1.25	[M+H]^+^	C_5_H_9_NO_2_	116.07061	−2.3	70.0651
15	D(+)-Pipecolinic acid^1^	1.25	[M+H]^+^	C_6_H_11_NO_2_	130.08626	0	130.0862
16	Quisqualic acid^1,2^	1.27	[M+H]^+^	C_5_H_7_N_3_O_5_	190.04585	0.6	144.0369, 83.0231, 57.0437
17	Maltose^1,2^	1.28	[M-H]^-^	C_12_H_22_O_11_	341.10894	0.8	341.1091, 179.0564, 161.0454, 119.0345, 89.0239, 71.0136, 59.0135
18	N-Acetyl-DL-phenylalanine^1^	1.42	[M+Na]^+^	C_11_H_13_NO_3_	230.07876	−3.6	130.0500, 84.0443
19	Fumaric acid^1,2^	1.43	[M-H]^-^	C_4_H_4_O_4_	115.00368	3.6	114.9313, 97.9336, 71.0149
20	Malic acid^1,2^	1.43	[M-H]^-^	C_4_H_6_O_5_	133.01425	1.9	115.0040, 71.0140
21	Adenosine^1,2,3^	1.56	[M+H]^+^	C_10_H_13_N_5_O_4_	268.10403	−0.3	136.0615, 119.0352
22	L-Pyroglutamic acid^1^	1.6	[M-H]^-^	C_5_H_7_NO_3_	128.03532	2.3	82.0301
23	N-Acetyl-L-glutamic acid^1^	1.72	[M-H]^-^	C_7_H_11_NO_5_	188.05645	2.7	168.8679, 102.0551, 78.9583
24	Guanosine^1,2^	1.89	[M+H]^+^	C_10_H_13_N_5_O_5_	284.09895	2.9	152.0555, 135.0291, 110.0355
25	Citric acid^1,2^	1.92	[M-H]^-^	C_6_H_8_O_7_	191.01973	2.9	111.0088, 87.0086, 67.0191, 57.0347
26	Sinapinaldehyde^1^	2.12	[M+H_2_O-H]^-^	C_11_H_12_O_4_	225.07575	3.1	177.0554, 148.0513, 134.0358, 91.0544, 77.0375
27	Gallic acid^1,2^	2.24	[M+Na]^+^	C_7_H_6_O_5_	166.08626	0.8	141.0670, 107.0133
28	D-Phenylalanine^1,2^	2.29	[M+H]^+^	C_9_H_11_NO_2_	166.08626	1.1	120.0808, 103.0542, 77.0389
29	Zeatin^1^	2.37	[M+Na]^+^	C_10_H_13_N_5_O	242.10123	−2.6	156.0623, 124.0755, 90.0546, 82.0642
30	Morroniside^1^	2.41	[M+Na]^+^	C_17_H_26_O_11_	429.13673	0.6	381.1110, 341.0134, 267.0803, 147.0647
31	2’-Hydroxy-4’,6’-dimethoxyacetophenone^1,2^	2.41	[M+H]^+^	C_10_H_12_O_4_	197.08084	0.7	153.0540, 137.0616, 105.0329, 91.0530, 77.0385
32	D-Pantothenic acid^1^	2.74	[M+H]^+^	C_9_H_17_NO_5_	220.11795	1.6	156.0623, 124.0755, 90.0546, 72.0444
33	Verbenalin^1^	2.78	[M+Na]^+^	C_17_H_24_O_10_	411.12617	0.9	182.0559, 137.0613
34	Protocatechuic acid^1^	2.87	[M+H]^+^	C_7_H_6_O_4_	155.03389	1.8	127.0525, 118.9193, 65.0384
35	Abscisic acid^1^	4.43	[M+H]^+^	C_15_H_20_O_4_	265.14344	2.5	195.1210, 172.0882, 152.0608, 129.0706, 91.0536
36	5,7-Dimethoxycoumarin^1^	4.81	[M+H]^+^	C_11_H_10_O_4_	207.06519	2.6	192.0422, 149.0225, 91.0546, 65.0385
37	L-Epicatechin^1^	4.87	[M+H]^+^	C_15_H_14_O_6_	291.08631	1.1	255.0630, 159.0379, 147.0400, 101.0350, 97.0283
38	Riboflavin^1^	5.13	[M+H]^+^	C_17_H_20_N_4_O_6_	377.14556	−3.5	319.1416, 243.0900, 198.0689
39	Gardenin B^1^	5.33	[M+H]^+^	C_19_H_18_O_7_	359.11253	−4.9	156.8933, 75.0484
40	Hydroxycinnamic acids^1^	5.48	[M-H]^-^	C_10_H_10_O_5_	209.04555	2.9	165.0555, 120.0210, 76.0317, 61.9882
41	Ellagic acid^1,2,3^	5.65	[M-H]^-^	C_14_H_6_O_8_	300.99899	−1.4	283.9955, 245.0099, 229.0141, 200.0111, 185.0241, 145.0303
42	Vanillin^1^	5.71	[M+H]^+^	C_8_H_8_O_3_	153.05462	1.6	93.0316, 65.0381
43	4-Hydroxy-3-methoxycinnamaldehyde^1^	6.07	[M+H]^+^	C_10_H_10_O_3_	179.06299	0.2	118.0412, 91.0539, 65.0383
44	N-lauryldiethanolamine^1^	6.65	[M+H]^+^	C_16_H_35_NO_2_	274.27406	2.2	274.2748, 256.2641, 88.0751, 70.0649
45	Phytosphingosine^1,2^	6.68	[M+H]^+^	C_18_H_39_NO_3_	318.30027	2.3	318.3009, 256.2635, 88.0751
46	3,3’-Di-O-methylellagic acid^1,2,3^	6.8	[M-H]^-^	C_16_H_10_O_8_	329.03029	0.5	314.0064, 298.9834, 270.9878, 242.9928
47	(Z)-5,8,11-Trihydroxyoctadec-9-enoic acid^1^	6.95	[M-H]^-^	C_18_H_34_O_5_	329.23335	0.1	229.1473, 211.1354, 183.1415, 171.1035
48	Ethanol,2,2’-(Tetradecylimino) bis^1^	7.1	[M+H]^+^	C_18_H_39_NO_2_	302.30536	3.1	284.2963, 106.0883, 88.0757, 70.0653
49	3,3’,4’-Tri-O-methylellagic acid^1,2^	7.53	[M-H]^-^	C_17_H_12_O_8_	343.04594	0.4	328.0223, 312.9984, 297.9751, 269.9801, 241.9856
50	Triphenylphosphine oxide^1^	7.54	[M+H]^+^	C_18_H_15_OP	279.09333	0.7	201.0476, 173.0534, 149.0236, 77.0379
51	Madecassic acid^1^	7.56	[M+H]^+^	C_30_H_48_O_6_	505.35237	0.7	469.3323, 451.2999, 405.3250, 145.0094
52	N,N-Diethyl-m-toluamide^1^	7.6	[M+H]^+^	C_12_H_17_NO	192.13829	0.8	119.0493, 91.0540, 65.0394
53	Hyocholic acid^1^	7.67	[M+H]^+^	C_24_H_40_O_5_	409.29485	0.8	409.3833
54	2alpha,19alpha,23-Trihydroxyoleanolic acid^1^	7.67	[M+H]^+^	C_30_H_48_O_6_	505.35237	0.7	469.3277, 439.3142, 395.2893, 189.1657
55	Quillaic acid^1^	7.69	[M+Na]^+^	C_30_H_46_O_5_	509.32375	0.5	235.1733, 189.1663
56	18β-Glycyrrhetinic acid^1^	7.95	[M+H]^+^	C_30_H_46_O_4_	471.34689	1.6	435.3278, 407.3202, 261.1835, 201.1656, 187.1501
57	Asiatic acid^1^	8.04	[M+H]^+^	C_30_H_48_O_5_	489.35745	1.1	407.3384, 203.1783, 175.1483, 145.1020
58	Phosphoric acid tris(2-chloro-1-methylethyl) ester^1^	8.35	[M+H]^+^	C_9_H_18_C_l3_O_4_P	327.00811	2.6	98.9838
59	15,17-Dihydroxy-12-octadecenoic acid^1^	8.49	[M-H]^-^	C_18_H_34_O_4_	313.23843	0.6	277.2175, 201.1147, 171.1033, 165.0922, 125.0972
60	Diphenylamine^1^	9.04	[M+H]^+^	C_12_H_11_N	170.09643	−0.2	93.0569
61	1-Palmitoyl-sn-glycero-3-phosphocholine^1,2^	9.23	[M+H]^+^	C_24_H_50_NO_7_P	496.33977	1.5	478.3286, 184.0737, 104.1069, 86.0968
62	1-Palmitoyl-sn-glycero-3-phosphoethanolamine^1^	9.24	[M+H]^+^	C_21_H_44_NO_7_P	454.29282	1.0	436.2786, 393.2490, 339.2904, 313.2752, 282.2802, 155.0111
63	1-Oleoyl-sn-glycero-3-phosphocholine^1^	9.24	[M+H]^+^	C_26_H_52_NO_7_P	522.35542	1.3	504.3453, 184.0741, 104.1072, 86.0963
64	Palmitic acid^1,2,3^	9.26	[M+H]^+^	C_16_H_32_O_2_	257.24751	2.0	89.0593, 69.0593, 57.0694
65	3,5,8-Trioxa-4-phosphahexacosa-17,20-dien-1-aminium,4-hydroxy-7-(hydroxymethyl)-N,N,N-trimethyl-9-oxo-, inner salt, 4-oxide, (17Z,20Z)- ^1^	9.43	[M+H]^+^	C_26_H_50_NO_7_P	520.33977	1.0	502.3296, 184.0736, 104.1063, 86.0961
66	Triphenyl phosphate^1^	9.46	[M+H]^+^	C_18_H_15_O_4_P	327.07807	0.6	251.0466, 215.0280, 152.0622, 98.9846
67	Traversianal^1^	9.68	[M+H]^+^	C_20_H_28_O_3_	317.21112	−4.0	245.2706, 183.0242, 159.0897, 109.1028
68	Germacrone^1^	9.69	[M+H]^+^	C_15_H_22_O	219.17434	2.2	219.1725, 163.1107, 135.0797, 91.0534
69	9(Z),11(E)-Octadecadienoic acid^1^	9.71	[M-H]^-^	C_18_H_30_O_3_	293.21222	0.5	249.2243, 195.1415, 177.1280, 167.1074, 139.1122, 113.0978
70	9,12-Octadecadiynoic acid^1^	9.76	[M+H]^+^	C_18_H_28_O_2_	277.21621	2.5	179.1446, 161.0948, 121.1010, 107.0853, 93.0700
71	6-Shogaol^1^	9.84	[M+H]^+^	C_17_H_24_O_3_	277.17892	2.8	235.1694, 179.1061, 131.0860, 57.0700
72	Echinulin^1^	9.84	[M+H]^+^	C_29_H_39_N_3_O_2_	462.31150	1.4	406.2471, 338.1866, 266.1914, 210.1278, 198.1286
73	Alpha-Cyperone^1^	9.93	[M+H]^+^	C_15_H_22_O	219.17434	2.2	163.1107, 135.0797, 111.0799
74	Cholesterol^1,2^	9.94	[M+Na]^+^	C_27_H_46_O	409.34409	2.6	409.3487, 391.2803, 137.1327, 95.0850, 81.0702
75	POPC^1^	9.96	[M+Na]^+^	C_42_H_82_NO_8_P	782.56703	1.0	723.4899, 599.4989, 577.5176, 184.0736, 86.0956
76	Leukotriene B5^1^	10.01	[M+H]^+^	C_20_H_30_O_4_	335.22169	−3.5	295.1971, 95.0850
77	Stigmasta-4,25-dien-3-one^1,2^	10.13	[M+H]^+^	C_29_H_46_O	411.36214	−0.2	393.3329, 327.2639, 271.2029, 109.0640, 97.0646
78	D-Camphor^1,2^	10.13	[M+H]^+^	C_10_H_16_O	153.12739	−0.5	135.1171, 91.0532, 77.0384, 69.0693, 55.0543
79	Oleic acid^1,2^	10.13	[M+H]^+^	C_18_H_34_O_2_	283.26316	2.1	149.1320, 135.1168, 121.1011, 93.0697, 81.0695, 69.0697, 55.0542
80	Clerosterol^1,2^	10.13	[M+H]^+^	C_29_H_48_O	413.37779	0.8	301.1350, 110.0714, 97.0645
81	2,4-Di-tert-butylphenol^1^	10.16	[M-H]^-^	C_14_H_22_O	205.15979	2.4	189.1291, 162.1127, 133.0302
82	17-Dihydroxy-12,14-octadecenoic acid^1^	10.19	[M-H]^-^	C_18_H_32_O_3_	295.22787	0.2	277.2184, 251.2394, 171.1040
83	Linolenic acid^1,2^	10.21	[M-H]^-^	C_18_H_30_O_2_	277.21730	1.2	149.0237, 95.0855, 81.0699, 67.0543
84	Stearic acid^1,2^	10.29	[M-H]^-^	C_18_H_36_O_2_	283.26425	1.7	265.1798, 221.1598
85	2,6-Di-tert-butyl-4-nitrophenol^1^	10.3	[M-H]^-^	C_14_H_21_NO_3_	250.14487	1.2	235.1224, 218.1205
86	12-Hydroxystearic acid^1^	10.38	[M-H]^-^	C_18_H_36_O_3_	299.25917	0.9	281.2480, 253.2537, 141.1279
87	β-Caryophyllene^1^	10.71	[M+H]^+^	C_15_H_24_	205.19508	3.3	205.1456, 149.0231, 121.0276, 93.0336, 65.0383
88	Acetyl tributyl citrate^1^	10.75	[M+H]^+^	C_20_H_34_O_8_	403.23264	2.1	259.1623, 185.0805, 157.0147, 139.0034, 129.0184, 111.0077
89	Lichesterylic acid^1^	10.85	[M-H]^-^	C_18_H_34_O_3_	297.24352	1.3	279.2358, 171.1044, 155.1096
90	20-HETE^1^	10.9	[M+H]^+^	C_20_H_32_O_3_	321.24242	−3.8	303.2233, 221.1766, 195.0933, 179.0954
91	Linoleic acid^1,2^	10.9	[M+H]^+^	C_18_H_32_O_2_	281.24751	3.3	147.1177, 133.1017, 119.0862, 95.0860, 81.0703, 55.0546
92	Cis-11-Eicosenoic acid^1^	11.03	[M-H]^-^	C_20_H_38_O_2_	309.27990	4.2	193.0889
93	Monostearin^1^	11.18	[M+H]^+^	C_21_H_42_O_4_	359.31559	0.5	341.3041, 123.1193, 95.0858, 71.0855, 57.0700
94	Patchouli alcohol^1^	11.31	[M+H]^+^	C_15_H_26_O	223.20564	1.0	190.9968, 149.0250, 124.0499, 111.0807, 57.0697
95	Kaurenoic acid^1^	11.32	[M+H]^+^	C_20_H_30_O_2_	303.23186	2.9	257.2273, 173.1329, 147.1169, 81.0699
96	Cis-13-Docosenoamide^1^	11.4	[M+H]^+^	C_22_H_43_NO	338.34174	1.7	321.3176, 303.3059, 149.1328, 135.1163, 83.0855
97	Myristic acid^1,2,3^	11.71	[M+H]^+^	C_14_H_28_O_2_	229.21621	3.7	103.0746, 89.0581, 69.0697, 57.0693
98	Heptadecanoic acid^1,2^	11.78	[M-H]^-^	C_17_H_34_O_2_	269.24860	2.7	269.2526
99	Arachidic Acid^1,2^	11.93	[M-H]^-^	C_20_H_40_O_2_	311.30283	3.4	228.2076, 61.9883
100	2-Hexadecenoic acid^1,2^	12.09	[M+H]^+^	C_16_H_30_O_2_	255.23186	1.6	149.1394, 135.1184, 79.0533, 69.0703, 55.0545
101	Laurocapram^1^	12.12	[M+H]^+^	C_18_H_35_NO	282.27914	1.4	149.1322, 135.1166, 121.1010, 83.0855, 69.0700
102	L-alpha-palmitin^1^	12.51	[M+H]^+^	C_19_H_38_O_4_	331.28429	2.0	313.2753, 109.1014, 95.0855, 71.0854, 57.0697
103	Vitamin D2^1^	12.71	[M+H]^+^	C_28_H_44_O	397.34649	3.5	315.1769, 172.9930, 158.9737, 70.0638
104	Pentadecanoic acid^1,2^	12.76	[M-H]^-^	C_15_H_30_O_2_	241.21730	2.7	225.0190, 181.1626, 151.0022
105	Glyceryl monooleate^1,2^	12.91	[M+H]^+^	C_21_H_40_O_4_	357.29994	1.0	339.2880, 265.2525, 247.2427, 149.1330, 135.1165, 121.1013
106	Pheophorbide A^1^	13.56	[M+H]^+^	C_35_H_36_N_4_O_5_	593.27585	0	533.2552

^1^Public database; ^2^Relevant literature; ^3^Reference standard.

#### 3.1.1 Identification of fatty acids

**Figure 2:**
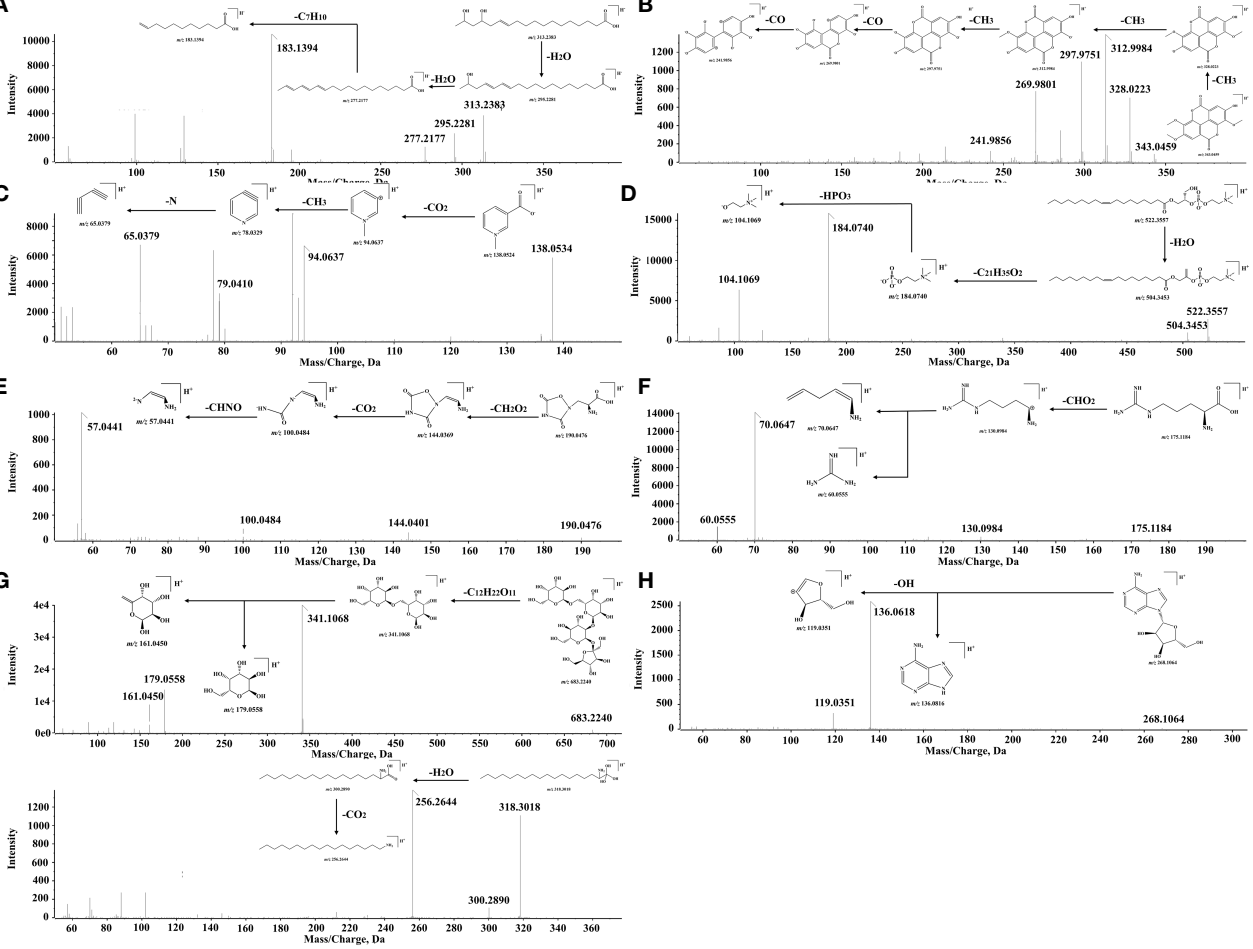
The cracking process of main compounds. **(A)** 15,17-Dihydroxy-12-octadecenoic acid. **(B)** 3,3’,4’-O-Trimethyl ellagic acid. **(C)** Trigonelline. **(D)** 1-Oleoyl-sn-glycero-3-phosphocholine. **(E)** Quisqualic acid. **(F)** L (+)-Arginine. **(G)** Stachyose. **(H)** Adenosine. **(I)** Phytosphingosine.

A total of 29 fatty acids and their derivatives were tentatively identified in QF, including 10 saturated fatty acids (48, 64, 84, 86, 93, 97–99, 102, and 104) and 19 unsaturated fatty acids (47, 59, 65, 67, 69, 70, 76, 79, 82, 83, 87, 89–92, 95, 96, 100, and 105). Take 15,17-dihydroxy-12-octadecenoic acid (constituent 59) for example to elucidate the cracking law of fatty acids. The precursor ion at *m/z* 313.2383 [M-H]^-^ was predicted to be the formula of C_18_H_34_O_4_, and the major MS/MS fragment ions were observed at *m/z* 295.2281 [M-H_2_O-H]^-^, 277.2177 [M-2H_2_O-H]^-^ and 183.1394 [M-2H_2_O-C_7_H_10_-H]^-^, respectively. It could be noted that the fragment ions of 295.2281 [M-H]^-^ and 277.2177 [M-H]^-^ were identified as 17-dihydroxy-12,14-octadecenoic acid (constituent 82) and linolenic acid (constituent 83) based on accurate mass weights and public database comparison. The process of cracking was illustrated in **Figure 2A**. Meanwhile, the fatty acids of constituent 64 (glutamic acid) and 97 (myristic acid) were identified based on reference standard and previous literature (Lu et al., 2014; Dai et al., 2024).

#### 3.1.2 Identification of organic acids and phenolic acids

A total of 26 organic acids and phenolic acids (6, 15, 19, 20, 25, 27, 31, 32, 34, 35, 37, 39–42, 46, 49, 53–57, 66, 71, 81, and 88) were initially characterized in QF. For instance, the formula of C_17_H_12_O_8_ was inferred according to the precursor ion at *m/z* 343.0459 [M-H]^-^, and the MS/MS fragment ions were exhibited at *m/z* 328.0223 [M-CH_3_-H]^-^, 312.9984 [M-2CH_3_-H]^-^, 297.9751 [M-3CH_3_-H]^-^, 269.9801 [M-3CH_3_-CO-H]^-^, and 241.9856 [M-3CH_3_-CO-H]^-^, respectively. The successive neutral losses of CH_3_ were easily produced, which may be attributed to the instability of methoxy on the benzene ring. Therefore, the fragment ions at *m/z* 343.0459 [M-H]^-^, 328.0223 [M-H]^-^, and 297.9751 [M-H]^-^ were ascribed as the 3,3’,4’-O-trimethyl ellagic acid (constituent 49), 3,3’-di-O-methylellagic acid (constituent 46), and ellagic acid (constituent 41), respectively. They were confirmed by the reference standards and the report of relevant literature (Zhang et al., 2015). The process of cracking is shown in **Figure 2B**.

#### 3.1.3 Identification of alkaloids and derivatives

A total of nine alkaloids (1, 4, 11, 29, 61–63, 75, and 85) were potentially determined in QF. For instance, the formula of C_7_H_8_NO_2_ was conjectured to be trigonelline (constituent 11) based on the precursor ion at *m/z* 138.0524 [M+H]^+^ and the major MS/MS fragment ions at *m/z* 93.0563 [M-CHO_2_+H]^+^, 79.0410 [M-CHO_2_-CH_2_-H]^+^, and 65.0379 [M-CHO_2_-CH_2_-N-H]^+^, respectively. It was eventually confirmed by the reference standard and literature (Wang et al., 2023). The proposed cleavage pathway is presented in **Figure 2C**. Constituents 61, 63, 65, and 73 were presumed to be the polyene phosphatidylcholine series constituents with a similar parent ion at *m/z* 184.0710 [M+H]^+^. The precursor ion at *m/z* 522.3557 [M+H]^+^ was considered to be the formula of C_26_H_52_NO_7_P. It was inferred as 1-Oleoyl-sn-glycero-3-phosphocholine with the MS/MS fragment ions at *m/z* 504.3453 [M-H_2_O+H]^+^, 184.0740 [M-H_2_O-C_21_H_35_O_2_+H]^+^, and 104.1069 [M-H_2_O-C_21_H_35_O_2_-HPO_3_+H]^+^, respectively. The above constituents were preliminarily determined through the public database comparison by characteristic fragments and previous literature (Song et al., 2023). The degradation process is described in **Figure 2D**.

#### 3.1.4 Identification of amino acids and derivatives

A total of 11 amino acids and derivatives (2, 3, 8, 9, 10, 14, 16, 18, 22, 23, and 28) were putatively observed in QF. For instance, the precursor ion at *m/z* 190.0476 [M+H]^+^ was deduced to be the formula of C_5_H_7_N_3_O_5_. It was inferred that the major MS/MS fragment ions of quisqualic acid were at *m/z* 144.0401 [M-CH_2_O_2_+H]^+^, 100.0484 [M-CH_2_O_2_-CO_2_+H]^+^, and 57.0441 [M-CH_2_O_2_-CO_2_-CHNO+H]^+^, respectively. It was consistent with the public database comparison and previous literature (Wang et al., 2023). The cracking rule is provided in **Figure 2E**. The precursor ion at *m/z* 175.1184 [M+H]^+^ was suspected to be the formula of C_6_H_14_N_4_O_2_. It was inferred to be L (+)-Arginine with the major MS/MS fragment ions at *m/z* 130.0984 [M-CHO_2_+H]^+^ and the cracked fragment ions at *m/z* 70.0647 [M+H]^+^ and 60.0555 [M+H]^+^. It was also confirmed by the public database comparison, reference standard, and previous literature (Liao, 2021). The fragmentation pathway is given in **Figure 2F**.

#### 3.1.5 Identification of glycosides

A total of 10 glycosides (5, 7, 12, 13, 17, 21, 24, 30, 33 and 51) were preliminarily revealed in QF. For instance, the precursor ion at *m/z* 683.2240 [M-H]^-^ was speculated to be the formula of C_24_H_44_O_22_. It was inferred as stachyose with the major MS/MS fragment ion at *m/z* 341.1068 [M-C_12_H_22_O_11_-H]^-^, which was cracked to the fragment ions at *m/z* 179.0558 [M-H]^-^ and 161.0450 [M-H]^-^ subsequently. The cracking law was in agreement with the public database comparison and previous literature (Li et al., 2023 ). The process of cleavage is shown in **Figure 2G**. The precursor ion at *m/z* 268.1064 [M+H]^+^ was believed to be the formula of C_10_H_13_N_5_O_4_. It was confirmed to be adenosine with the major MS/MS fragment ion at *m/z* 136.0618 [M+H]^+^ and 119.0351 [M+H]^+^, which was the same as the database comparison and reference standard. The cracking rule is illustrated in **Figure 2H**.

#### 3.1.6 Identification of others

A total of 21 other constituents (26, 36, 38, 43–45, 50, 52, 58, 60, 68, 72–74, 77, 78, 80, 94, 101, 103, and 106) were tentatively presumed in QF. For instance, the precursor ion at *m/z* 318.3018 [M+H]^+^ was considered to be the formula of C_18_H_39_NO_3_. It was inferred as phytosphingosine with the major MS/MS fragment ion at *m/z* 300.2090 [M-H_2_O+H]^+^, which was cracked to the fragment ion at *m/z* 256.2644 [M-H_2_O-CO_2_+H]^+^ due to the unstable enol structure. It was identified through database comparison by characteristic fragment ions and previous literature (Liu et al., 2014). The cracking process is described in **Figure 2I**. Moreover, other constituents were also preliminarily identified through public database comparison by the characteristic fragments and previous literature (Wen et al., 2020).

### 3.2 Chemometric analysis

**Figure 3:**
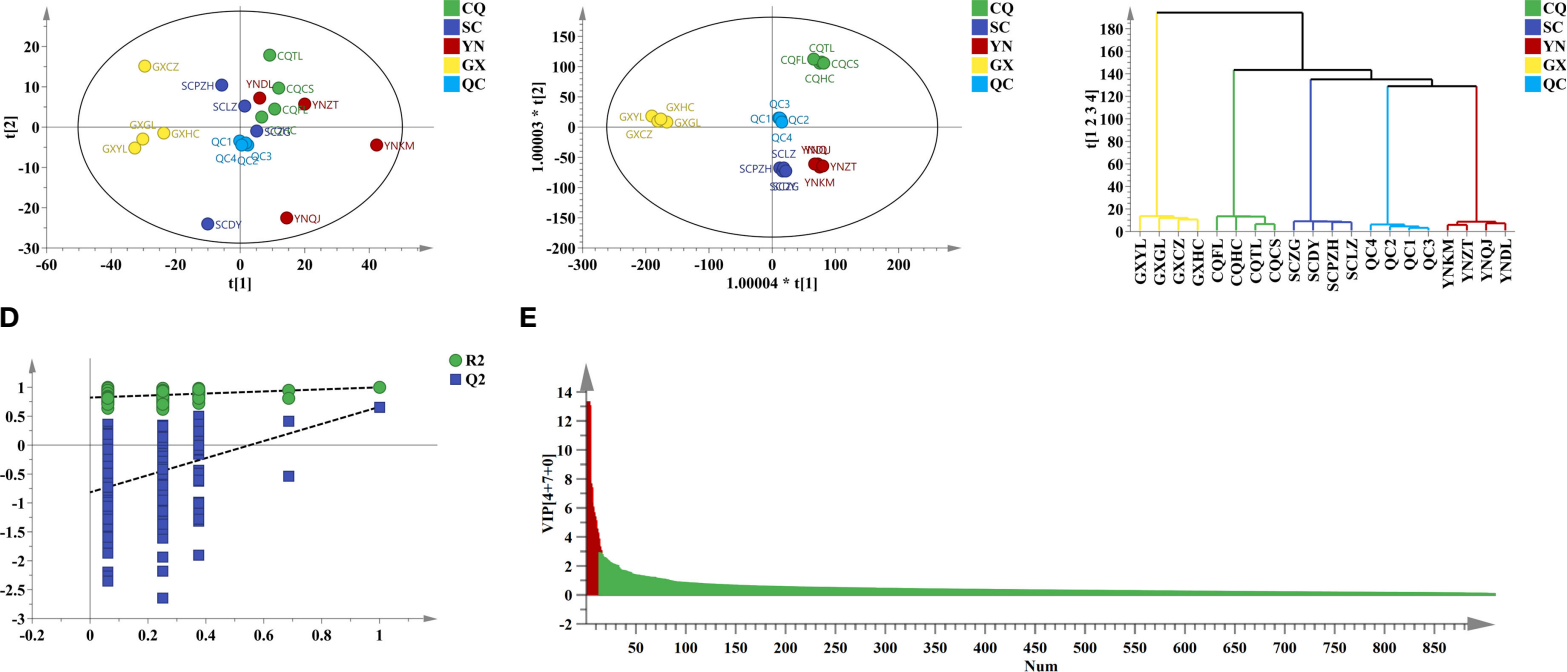
Chemometric analysis of QF from different origins in pos. **(A)** PCA score plot. **(B)** OPLS-DA score plot. **(C)** HCA score plot. **(D)** Two hundred times permutation. **(E)** VIP value.

**Figure 4:**
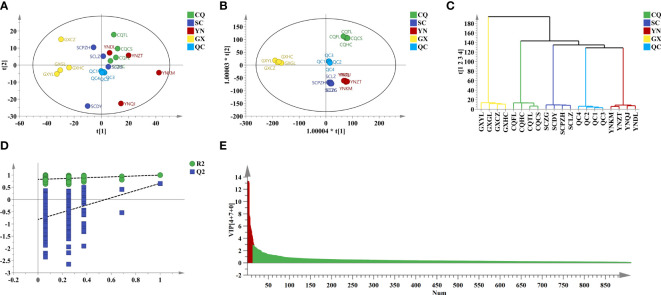
Chemometric analysis of QF from different origins in neg. **(A)** PCA score plot. **(B)** OPLS-DA score plot. **(C)** HCA score plot. **(D)** Two hundred times permutation. **(E)** VIP value.

To screen potential Q-markers and compare geographical differences of QF from different origins, chemometric analysis was performed for further analysis. The converted data including 2,552 variables in positive ion mode and 912 variables in negative ion mode were employed for principal component analysis (PCA), which was an unsupervised recognition mode and could observe the distribution trend of samples through data downscaling. Model parameters of PCA in positive (*R* ^2^ *X* = 0.649, *Q* ^2^ = 0.266) and negative (*R* ^2^ *X* = 0.802, *Q* ^2^ = 0.421) modes were relatively poor. Cumulative variance contribution rate (PC1 and PC2) explained 24.6% and 17.1% in positive ion mode, and explained 36.9% and 12.1% in negative ion mode, respectively. The score plot of PCA (**Figures 3A**, **4A**) showed that the distinction between CQ, SC, and YN samples was ambiguous except for GX samples.

To amplify the differences between groups and visual presentation, orthogonal partial least squares discriminant analysis (OPLS-DA) was applied subsequently to better-distinguished origins. The model parameters of OPLS-DA in positive (*R* ^2^ *X* = 0.926, *R* ^2^ *Y* = 0.997, and *Q* ^2^ = 0.740) and negative (*R* ^2^ *X* = 0.975, *R* ^2^ *Y* = 0.995, and *Q* ^2^ = 0.604) ion modes were greater than 0.5, which indicated that the reliability and predictability of the model were well (Gao et al., 2023). The score plot of OPLS-DA (**Figures 3B**, **4B**) showed that the QF from different origins could be divided into four categories based on geographical resources. The distinction of hierarchical cluster analysis (HCA) presented in **Figures 3C**, **4C** was more obvious and intuitive, which was consistent with the result of OPLS-DA. The validity of the model was evaluated by 200 permutation tests (**Figures 3D**, **4D**). The model parameter of 200 permutation tests in positive (*R* ^2^ = 0.927, *Q* ^2^ = −0.432) and negative (*R* ^2^ = 0.822, *Q* ^2^ = −0.817) ion modes indicated that the model was not over-fitting.

Potential Q-makers in QF were screened by variable importance for projection (VIP). The higher the VIP score (**Figures 3E**, **4E**) of the constituents presented, the more relevant to origin distribution. A total of 16 components were screened with a VIP score > 3 and *p*-value > 0.05, as presented in **Table 2**. Among them, the fatty acids including oleic acid, palmitic acid, linoleic acid, linolenic acid, myristic acid, glyceryl monooleate, and 17-dihydroxy-12,14-octadecenoic acid were the common constituents in plants with the various pharmacological activities including cardiovascular disease and anti-inflammatory and immunomodulatory effect (Shayan et al., 2020; Coniglio et al., 2023). The polysaccharides including stachyose and maltose have the effects of regulating gut microbiota and liver protection (Cui et al., 2021; Xu et al., 2022). The polyene phosphatidylcholine including 1-oleoyl-sn-glycero-3-phosphocholine and POPC could ameliorate synovial inflammation and acute liver injury (Sun et al., 2023; Yin et al., 2023). As the characteristic components in QF, trigonelline, quisqualic acid, and 3,3’-di-O-methylellagic acid had several pharmacological activities including antidiabetic effects, neural paralysis, anticancer, and others (Ranger et al., 2011; Rad et al., 2022; Liang et al., 2023). They could be widely used for quality evaluation in the future. The rich pharmaceutical active ingredients in QF reflected the enormous development prospects in TCM discovery.

**Table 2:** Screening potential quality markers in different origins of QF.

No.	Constituent name	Rt (min)	Ion type	Observed *m/z*	VIP
3	L (+)-Arginine	1.16	[M+H]+	175.11895	4.11492
7	Stachyose	1.22	[M-H]^-^	683.22520	5.12873
11	Trigonelline	1.24	[M+H]^+^	138.05510	3.03881
16	Quisqualic acid	1.27	[M+H]^+^	190.04590	3.78323
17	Maltose	1.28	[M-H]^-^	341.10890	5.72571
46	3,3’-Di-O-methylellagic acid	6.80	[M-H]^-^	329.03030	3.12424
63	1-Oleoyl-sn-glycero-3-phosphocholine	9.24	[M+H]^+^	522.35540	6.05674
64	Palmitic acid	9.26	[M+H]^+^	257.24751	3.46212
75	POPC	9.96	[M+Na]^+^	782.56703	5.06767
77	Stigmasta-4,25-dien-3-one	10.13	[M+H]^+^	411.36214	6.75580
79	Oleic acid	10.13	[M+H]^+^	283.26316	10.0554
82	17-Dihydroxy-12,14-octadecenoic acid	10.19	[M-H]^-^	295.22787	3.71475
83	Linolenic acid	10.21	[M-H]^-^	277.21730	5.53362
91	Linoleic acid	10.90	[M+H]^+^	281.24751	5.67898
97	Myristic acid	11.71	[M+H]^+^	229.21621	4.98823
105	Glyceryl monooleate	12.91	[M+H]^+^	357.29994	6.06811

### 3.3 Quantitative analysis of QF from different origins

#### 3.3.1 Condition optimization

In this study, the extraction method (ultrasonication and reflux), extraction time (15 min, 30 min, 45 min, and 60 min), extraction solvent (water and 25%, 50%, 80%, and 100% methanol), and solvent–sample ratios (10:1, 20:1, 35:1, and 50:1) were investigated. It was found that 0.5 g of QF sample powder in 5 mL of 80% methanol was ultrasonically extracted for 30 min with the advantages of easy extraction, smooth chromatogram baseline, and high response of each common peak. Therefore, the above conditions were determined as the method to prepare the test solutions.

The amino column was employed to detect the content of trigonelline under the content determination in the Chinese Pharmacopoeia (China Pharmacopoeia [Part I], 2020). However, the stability and durability of amino columns are poor and not widely used. Relevant literature reported that trigonelline was an amphoteric compound, which was not retained on C18 columns. Therefore, ion-pairing reagents were added to increase its retention time on C18 columns (Arai et al., 2015). In addition, flow rates (0.8 mL/min, 1.0 mL/min, and 1.2 mL/min), column temperatures (25°C, 30°C, and 35°C), and acetonitrile and methanol with different modifiers (0.05%, 0.1%, 0.2% phosphoric acid, 4, 6, 8, 10 mmol/L sodium 1-octane sulfonate, 10 mmol/L sodium dodecyl sulfonate, and 0.2 mmol/L ammonium chloride) were optimized. The results indicated that the shape of chromatographic peaks and the separation were better when the mobile phase is as follows: In acetonitrile–10 mmol/L sodium 1-octanesulfonate with 0.1% phosphoric acid, the flow rate was 1.0 mL/min and the column temperature was 30°C. Gradient elution conditions and equilibration time before sample injection were optimized at the same time, the details as shown in Section 2.5.

**Figure 5:**
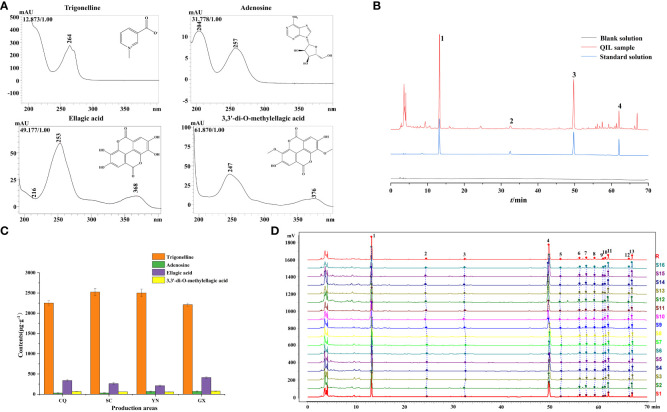
**(A)** The maximum absorption wavelengths. **(B)** The HPLC chromatograms of QF. (1) Trigonelline; (2) adenosine; (3) ellagic acid; (4) 3,3’-di-O-methylellagic acid. **(C)** The contents of target constituents in QF from different origins. **(D)** HPLC fingerprints of 16 batches QF.

The maximum absorption wavelengths of trigonelline, adenosine, ellagic acid, and 3,3’-di-O-methylellagic acid were 264 nm, 257 nm, 254 nm, and 247 nm, respectively, as depicted in **Figure 5A**. All of them were compared with the maximum absorption wavelength and retention time of the corresponding reference standards, which were also consistent with the results of UPLC-QTOF-MS analysis. As the wavelength at 254 nm performed a higher response of each chromatographic peak by comparing with other wavelengths, it was selected to be the detection wavelength of HPLC.

#### 3.3.2 Method validation

According to the Guidelines of Analytical Methods Validation in Chinese Pharmacopoeia (China Pharmacopoeia [Part I], 2020), the specificity, linearity, limit of detection (LOD), limit of quantitation (LOQ), precision, stability, repeatability, and recovery were evaluated to confirm the reliability of the established HPLC method. The HPLC chromatograms of sample S1 are demonstrated in **Figure 5B** for specificity. In the linearity test, the mixture of standard solutions of different concentrations was determined to establish regression equations, which were calculated by the abscissa *X* of concentrations (*x*, μg/mL) and ordinate *Y* of peak areas (*y*). Additionally, the signal-to-noise ratios of 10 and 3 were defined individually as the LOQ and LOD, which were evaluated by diluting the mixture of standard solutions successively. The appropriate mixture of standard solutions was consecutively analyzed six times for precision tests. The same S1 sample after being stored at room temperature for 0 h, 2 h, 4 h, 8 h, 12 h, and 24 h were analyzed for stability tests. Six samples of the same solution preparation method were analyzed for repeatability tests. Meanwhile, the four reference standards equivalent to 100% of the sample S1 content were added individually into the six sample S1 for recovery.

In summary, the results indicated that the correlation coefficients (*r*) were greater than 0.9997. The relative standard deviations (RSDs) of precision, stability, and repeatability were less than 1.5%, and the recoveries were in the range of 98.8%–103.3%, which indicated that the above method was reliable, as summarized in **Table 3**.

**Table 3:** The results of HPLC methodology validation.

Constituent name	Regression equation	*r*	Linearity range (μg/mL)	LOD (μg/mL)	LOQ (μg/mL)	Precision (RSD, %)	Stability (RSD, %)	Repeatability (RSD, %)	Recovery RSD (%, *n*=6)
Trigonelline	*y* = 23,639*x−*272	0.9999	22.4–224	0.055	0.180	0.94	1.16	1.25	102.0 ± 1.33
Adenosine	*y* = 65,653*x−*1469	0.9999	0.822–8.22	0.028	0.093	0.62	0.59	1.00	103.3 ± 1.50
Ellagic acid	*y* = 18,4971*x*+2966	0.9999	4.12–41.2	0.003	0.009	0.36	1.06	1.44	101.7 ± 1.94
3,3’-Di-O-ethylellagic acid	*y* = 158,789*x−*7999	0.9997	0.800–8.00	0.004	0.015	0.73	0.58	1.17	98.8 ± 2.19

#### 3.3.3 Quantification of four target constituents

Each batch of QF powder (0.5 g) was prepared according to Section 2.3 conditions and determined according to Section 2.5 conditions. The content of four target constituents in QF was calculated through the peak area by the calibration curves. The results of content determination showed that the content of trigonelline (2,165–2,615 μg/g) and 3,3’-di-O-methylellagic acid (52.69–79.79 μg/g) in QF varied little among the different origins. It also indicated that the content of trigonelline met the requirement of Chinese Pharmacopeia (2020 edition) and was consistent with the previous report (Wen et al., 2020), while the content of adenosine (15.92–84.52 μg/g) and ellagic acid (189.3–434.8 μg/g) in QF varied greatly among the different origins. It could be observed from **Figure 5C** that the average content of adenosine in QF from the origins of YN (66.19 μg/g) and GX (70.30 μg/g) were significantly higher than the origins from CQ (28.29 μg/g) and SC (29.38 μg/g). The highest and lowest content of ellagic acid were found in the origins of GX (413.3 μg/g) and YN (210.1 μg/g), respectively. Meanwhile, many research has found that the above constituents were rich in pharmacological properties at the appropriate dose, such as treating memory impairment (Aktar et al., 2024), attenuating neuroinflammation (Liu et al., 2023), and immunomodulatory (Zhang et al., 2022) and antioxidant (Mohamed et al., 2018) properties. The above result of content determination provided reference for the quality evaluation and drug application.

#### 3.3.4 HPLC fingerprints analysis

HPLC fingerprints of QF were obtained by introducing the raw data of 16 batch samples into the “Similarity Evaluation System for Chromatographic Fingerprint of Traditional Chinese Medicine (2012 Edition)” software in AIA format. A total of 13 peaks were matched as the common constituents after setting the 0.2 widths of the time window, matching automatically and taking S1 as the reference spectrum (R). Moreover, the peak 1 (Trigonelline), peak 3 (Adenosine), peak 4 (Ellagic acid), and peak 11 (3,3’-di-O-methylellagic acid) were identified based on reference standard comparison and maximum absorption wavelengths. The HPLC fingerprints of 16 batches of QF are shown in **Figure 5D**. In addition, peak 1 (Trigonelline) was set as the reference peak to evaluate the reliability of the method and conditions. It showed that the RSDs of the retention time (RT) and average peak area of the other 12 common peaks were less than 3%, indicating that the method was admitted with perfect precision, accurate repeatability, and stable test solution.

The results of the similarity evaluation are provided in **Table 4**. The similarity between 16 batches of QF was in the range of 0.870–0.999, indicating that the active compounds of QF from different origins were extremely similar. The established HPLC fingerprint method of QF could be used for quality consistency evaluation and species identification in the future.

**Table 4:** The results of similarity evaluation between different batches of QF.

	S1	S2	S3	S4	S5	S6	S7	S8	S9	S10	S11	S12	S13	S14	S15	S16	R
S1	1.000																
S2	0.999	1.000															
S3	0.990	0.991	1.000														
S4	0.996	0.997	0.998	1.000													
S5	0.989	0.990	0.998	0.996	1.000												
S6	0.977	0.979	0.996	0.991	0.997	1.000											
S7	0.995	0.996	0.997	0.998	0.998	0.991	1.000										
S8	0.997	0.998	0.982	0.991	0.982	0.966	0.991	1.000									
S9	0.966	0.964	0.922	0.942	0.918	0.890	0.939	0.974	1.000								
S10	0.986	0.983	0.955	0.969	0.950	0.930	0.966	0.988	0.995	1.000							
S11	0.992	0.991	0.966	0.979	0.964	0.944	0.977	0.996	0.989	0.996	1.000						
S12	0.993	0.991	0.967	0.979	0.964	0.945	0.977	0.994	0.990	0.999	0.999	1.000
S13	0.970	0.968	0.929	0.947	0.926	0.900	0.946	0.978	0.998	0.996	0.990	0.991	1.000
S14	0.953	0.951	0.904	0.926	0.990	0.870	0.924	0.964	0.998	0.989	0.982	0.982	0.997	1.000			
S15	0.970	0.968	0.929	0.947	0.925	0.990	0.946	0.977	0.998	0.997	0.990	0.992	0.999	0.997	1.000		
S16	0.995	0.995	0.999	0.999	0.997	0.993	0.998	0.988	0.936	0.964	0.975	0.976	0.941	0.919	0.941	1.000	
R	1.000	0.999	0.987	0.994	0.985	0.972	0.993	0.998	0.972	0.989	0.995	0.995	0.976	0.961	0.976	0.992	1.000

## 4 Conclusion

In this study, an accurate and systematic UPLC-Q-TOF-MS approach was first established to characterize the alcohol–aqueous soluble constituents of QF from different origins. A total of 106 constituents were tentatively identified through reference standards, public database comparison, and previous literature, namely, 29 fatty acids, 26 organic acids, 11 amino acids and derivatives, 10 glycosides, 9 alkaloids and derivatives, and 21 other compounds. Among them, a total of 68 constituents, 30 constituents, and 8 constituents were characterized through database matching, previous report, and reference standards, respectively. The chemometric analysis was utilized to screen potential Q-markers and compare the differences in the geographical origin of QF. Eventually, QF from different origins were effectively distinguished and 16 components were screened as the important differential markers.

In addition, an effective and convenient reversed-phase HPLC method was established to simultaneously determine four target constituents in QF. Meanwhile, it was confirmed that the analytical method was reliable in terms of linearity, precision, stability, repeatability, and recovery. The HPLC fingerprint of QF further proved that the common constituents of 16 batches of QF were extremely similar, and the similarity was in the range of 0.870–0.999. The above research provides some insights for the research on the pharmacodynamic constituents, quality control, and origin identification of QF. It also lays a scientific basis for the effective utilization and development of QF.

## Data availability statement

The original contributions presented in the study are included in the article/supplementary material. Further inquiries can be directed to the corresponding author.

## Author contributions

DH: Funding acquisition, Supervision, Writing – review & editing. LD: Conceptualization, Data curation, Writing – original draft. LY: Conceptualization, Data curation, Writing – original draft. WQ: Formal analysis, Project administration, Writing – review & editing. YW: Formal analysis, Project administration, Writing – review & editing. JZ: Investigation, Methodology, Writing – review & editing. SP: Investigation, Methodology, Writing – review & editing.
