# Corrigendum: Chemical constituent characterization and determination of *Quisqualis fructus* based on UPLC-Q-TOF-MS and HPLC combined with fingerprint and chemometric analysis

**DOI:** 10.3389/fpls.2024.1463256

**Published:** 2024-08-13

**Authors:** Lin Yang, Lei Dai, Weihan Qin, Yiwu Wang, Jianing Zhao, Shuxiang Pan, Dan He

**Affiliations:** ^1^ Chongqing Pharmaceutical Preparation Engineering Technology Research Center, Chongqing Medical and Pharmaceutical College, Chongqing, China; ^2^ Chongqing Research Center for Pharmaceutical Engineering, College of Pharmacy, Chongqing Medical University, Chongqing, China; ^3^ Medicinal Chemistry Institute of Traditional Chinese Medicine, Chongqing Academy of Chinese Material Medica, Chongqing, China

**Keywords:** *Quisqualis fructus*, UPLC-Q-TOF, HPLC, chemometric, fingerprint

In the published article, there was an error in the **Funding** statement. The first funding number was wrong. “This research was supported by grants from the Research Program of Chongqing Municipal Education Commission (KJZDK202302804) and Chongqing Graduate Tutor Team Construction Project of Chongqing Municipal Education Commission Foundation (cqmudstd202216)”. The correct Funding statement appears below.

“This research was supported by grants from the Research Program of Chongqing Municipal Education Commission (KJQN202302814) and Chongqing Graduate Tutor Team Construction Project of Chongqing Municipal Education Commission Foundation (cqmudstd202216).”

The authors apologize for this error and state that this does not change the scientific conclusions of the article in any way. The original article has been updated.

